# The association between diabetes mellitus and HRQoL of older people in Shanghai

**DOI:** 10.1186/s12877-021-02590-3

**Published:** 2021-11-03

**Authors:** Shiyin Tian, Rui Wang, Mengxing Qian, Lijuan Liu, Zhenyi Shao, Cheng Wu, Jinhai Sun

**Affiliations:** 1 Department of Health Management, Navy military medical university, Shanghai, China; 2 Medical Department, Shanghai Changzheng Hospital, Shanghai, China; 3 Department of Health Statistics, Navy Military Medical University, Shanghai, China; 4 Centre for Health Statistics and Information of Shanghai, Shanghai, China

**Keywords:** Diabetes mellitus, Quality of life, Older people

## Abstract

**Background:**

This study aimed to explore the association between diabetes mellitus and health-related quality of life (HRQoL) of older people in Shanghai, China, especially regarding the differences in each aspect of the EQ-5D and how large the score gaps are between older people with and without diabetes.

**Methods:**

A total of 11,103 people of either sex older than 60 years were enrolled from 17 districts of Shanghai. The EQ-5D-3L was used to assess the HRQoL of older people. The Wilcoxon rank sum test and t-test were used to compare the difference in HRQoL between people with or without diabetes. After univariate regression, multiple linear regression and ordinal logistic regression were conducted to evaluate the influence of diabetes mellitus and other confounding variables on the EQ VAS scores and on the five dimensions of the EQ-5D.

**Results:**

Twelve percent of all participants had diabetes mellitus, and the proportion was almost the same between men and women. The EQ VAS scores of people with diabetes mellitus were approximately 3.70 points lower than those of people without diabetes (95% CI = -4.40,  -2.99, *p* < 0.001) after adjusting for confounding variables. People with diabetes mellitus had increased problems with mobility (OR = 1.57, 95% CI = 1.33, 1.85), self-care (OR = 1.65, 95% CI = 1.35, 2.01), usual activities (OR = 1.78, 95% CI = 1.51, 2.11), pain/discomfort (OR = 1.42, 95% CI = 1.24, 1.64), and anxiety/depression (OR = 1.33, 95% CI = 1.07, 1.64).

**Conclusions:**

This study showed that diabetes mellitus was associated with the HRQoL of older people and that older people with diabetes had poorer performance in every aspect of EQ-5D measurements.

## Backgrounds

Many countries have a rapidly ageing population, and the global population of people aged 60 years and older will more than double, from 900 million in 2015 to approximately 2 billion, in 2050 [1]. In China, according to the latest report released by the China Development Research Foundation in June 2020, individuals over 65 will make up 14% of the total population in approximately 2022 and reach 27.9% in 2050. With the growing number of older people, a surge in the prevalence and incidence of age-associated diseases is inevitable [2]. In addition, older people often more frequently experience poor functional health and are more vulnerable to diseases [3]; hence, the quality of life of the geriatric population is raising increased concern.

Among the many age-associated diseases, diabetes mellitus is one of the most common chronic diseases among older people. The number of people with diabetes rose from 108 million in 1980 to 422 million in 2014 worldwide [4]. People who have diabetes are at a high risk of many complications, such as low-grade inflammation, cardio-cerebrovascular disease and amputation [5], and these complications are sometimes atypical and can take a long time to discover [6], which has more serious negative consequences for the health of geriatric people with diabetes. Additionally, the treatment of diabetes encompasses adherence to strict eating guidelines, regular use of antidiabetic drugs and/or insulin, self-monitoring of blood glucose and other long-term treatments [7], all of which influence patient quality of life.

Among the many methods for assessing quality of life, health-related quality of life (HRQoL) is used worldwide and is an important way to measure the impact of chronic disease on people’s quality of life [8]; moreover, HRQoL is often used to study the health status of older people [9]. Thus, in this article, we employed HRQoL to measure the association between diabetes mellitus and the quality of life of older people.

Previous studies have identified several chronic diseases that are associated with HRQoL. A study of the relationship between chronic diseases and HRQoL showed that older adults (> 65 years) with dementia/Alzheimer’s disease, stroke/transient ischaemic attack (TIA), osteoarthritis/gout/rheumatoid arthritis (RA), and osteoporosis had a lower HRQoL [10], and another study found that depression, stroke, heart disease and cognitive dysfunction had a significantly adverse impact on HRQoL [11]. In a systematic review in 2018, ten articles studied the relationship between diabetes mellitus and HRQoL, but the study with the highest values was conducted on participants with a younger mean age (57.2 years) [12]. There is little literature about how diabetes mellitus influences HRQoL in the geriatric population, and our study aimed to fill this research gap by focusing on the HRQoL of older people with diabetes mellitus.

## Methods

### Study design and data source

The aim of the study was to explore the association between diabetes mellitus and health-related quality of life (HRQoL) of older people in Shanghai, especially regarding the differences in each aspect of the EQ-5D and how large the score gaps are between older people with and without diabetes. All older people with diabetes mellitus in this study were diagnosed with diabetes by a doctor.

The data used in this study were extracted from the fifth National Household Health Survey of Shanghai, China (NHHS), which was conducted in 2013 [13]. The NHHS is a cross-sectional study organised by the National Health Commission of China every five years, and the data were collected by a face-to-face interview conducted by trained staff. The households were sampled using a multistage stratified cluster sampling procedure. Informed consent forms were provided and signed before the interview.

### Participants and measurement

A total of 11,103 older people of either sex aged over 60 years were enrolled from 17 districts of Shanghai, China.

The EQ-5D-3L was used to assess the HRQoL of older people. The EQ-5D consists of 2 parts: the EQ-5D descriptive system and the EQ visual analogue scale (EQ VAS). The EQ-5D descriptive system comprises five dimensions: mobility, self-care, usual activities, pain/discomfort and anxiety/depression; each dimension has 3 levels: no problems, some problems, and extreme problems. The interviewees were asked to describe their health status for each of the five dimensions with the three levels. The EQ VAS is an analogue scale where the endpoints are labelled “Best imaginable health state”, worth 100 points, and “Worst imaginable health state”, worth 0 points. Respondents were asked to self-rate their health between 0 and 100 points. The EQ VAS can be used as a quantitative measure of health outcomes that reflects the patient’s own judgement of their health [14]. The Chinese version of the EQ-5D questionnaire has been demonstrated to assess quality of life [15], and the reliability of the EQ-5D was good in this study (Cronbach’s alpha = 0.841).

Diabetes mellitus was assessed by the interviewer with the question “Have you been diagnosed with diabetes mellitus by a doctor?”, and the outcome of the question was a binary variable that indicated whether the subject had diabetes mellitus. All older people with diabetes mellitus in this study were aware of their disease and were diagnosed by a doctor.

According to previous studies, several influencing factors should be considered. Body mass index (BMI) groups had a close relationship with HRQoL, so BMI was considered an influencing factor. Additionally, two types of control variables were collected, one of which was sociodemographic variables including age, sex, registered residence, education, marital status, and income source (the classification are shown in Table 1), and the other was health-related behaviours such as smoking, drinking, physical exercise and health examination participation. Smoking status was divided into three levels: everyday smoking, not everyday smoking, and never smoking. Drinking represented whether the participant had consumed alcohol in the previous 12 months. Physical exercise referred to whether the participant had exercised in the past 6 months, and health examination indicated whether the subject had his/her health checked in the past year.

**Table 1:** General characteristics of older people according to sex

Characteristics	Male N (%)	Female N (%)	Total N (%)
Age			
60–69	3002(57.2)	3144(53.7)	6146(55.4)
70–79	1436(27.4)	1588(27.1)	3024(27.2)
≥80	806(15.4)	1127(19.2)	1933(17.4)
Registered residence			
Rural	977(18.6)	1212(20.7)	2189(19.7)
Urban	4267(81.4)	4647(79.3)	8914(80.3)
BMI (kg/m^2^)			
< 18.5	300(5.7)	476(8.1)	776(7.0)
18.5–23.9	2950(56.3)	3277(55.9)	6227 (56.1)
24–27.9	1707(32.6)	1674(28.6)	3381(30.5)
≥28	287(5.5)	432(7.4)	719(6.5)
Education			
Illiterate	407(7.8)	1458(24.9)	1865(16.8)
Primary school	1353(25.8)	1639(28.0)	2992(26.9)
Junior high school	1643(31.3)	1473(25.1)	3116(28.1)
Senior high school and above	1841(35.1)	1289(22.0)	3130(28.2)
Marital status			
Single	82(1.6)	76(1.3)	158(1.4)
Widowed	484(9.2)	1579(26.9)	2063(18.6)
Divorced	52(1.0)	81(1.4)	133(1.2)
Married	4626(88.2)	4123(70.4)	8749(78.8)
Income source			
Oneself or mate	4990(95.2)	5413(92.4)	10,403(93.7)
Children or grandchildren	146(2.8)	301(5.1)	447(4.0)
Social relief	48(0.9)	76(1.3)	124(1.1)
Other	60(1.1)	69(1.2)	129(1.2)
Smoking			
Never	3261(62.2)	5741(98.0)	9002(81.1)
Not everyday	201(3.8)	64(1.1)	265(2.4)
Everyday	1782(34.0)	54(0.9)	1836(16.5)
Drinking			
Yes	1764(33.6)	154(2.6)	1918(17.3)
No	3480(66.4)	5705(97.4)	9185(82.7)
Physical exercise			
Yes	2982(56.9)	3102(52.9)	6084(54.8)
No	2262(43.1)	2757(47.1)	5019(45.2)
Health examination			
Yes	3212(61.3)	3497(59.7)	6709(60.4)
No	2032(38.7)	2362(40.3)	4394(39.6)
Diabetes mellitus			
Yes	671(12.8)	745(12.7)	1416(12.8)
No	4573(87.2)	5114(87.3)	9687(87.2)

### Statistical analysis

Descriptive statistics were employed to describe the general characteristics of the 11,103 older people. Categorical data and ranked data are presented as numbers (percentages). Continuous variables are presented as the mean ± SD. The proportions of the three levels (no problems, some problems, and extreme problems) reported for the five dimensions of the EQ-5D were also described separately for older people with diabetes mellitus and those without; the Wilcoxon rank sum test was performed to compare the differences in each of the five dimensions of the EQ-5D between the people with or without diabetes, and a t-test was used to compare EQ VAS scores between the two groups.

Univariate analysis was conducted to assess the effects of every characteristic on diabetes. A multiple linear regression model was conducted to evaluate the associations of diabetes mellitus with other confounding variables and EQ VAS scores. Multivariate model 1 was adjusted for age, sex, registered residence, BMI, education, marital status, income source, smoking, drinking, physical exercise and health examination. Model 2 was adjusted for all model 1 factors plus hypertension and other chronic diseases. Ordinal logistic regression was conducted to evaluate the associations of diabetes mellitus with the five dimensions of the EQ-5D and adjusted for all the influencing variables in model 2.

BMI groups, marital status and income source were coded as dummy variables. Less than 5% of the participants had missing data across all the variables. The missing data were imputed to the dominant category for categorical variables and to the mean for continuous variables.

All analyses were conducted using SPSS software, version 22.0 (IBM Corporation, Chicago, IL, USA) with statistical significance set at a *P* value < 0.05, and the figure was generated using GraphPad Prism version 7.00 (GraphPad Software, San Diego, CA, USA).

## Results

### Characteristics of the study participants

The characteristics of all participants are shown in Table 1 and are also presented separately according to sex. A total of 12.8% of all subjects had diabetes mellitus, and the proportions of males and females were 12.8 %and 12.7%, respectively. Moreover, 55.4% of all the older people were aged 60 to 69 years old, 27.2% were aged 70 to 79, and 17.4% were aged above 80 years old.

### The HRQoL of people with or without diabetes mellitus

The HRQoL scores of older people with or without diabetes mellitus are presented in Table 2. The proportions of people with no problems across the five dimensions— mobility, self-care, usual activities, pain/discomfort and anxiety/depression—were 86.3%, 92.0%, 88.1%, 80.6% and 93.2%, respectively, and the mean EQ VAS score was 76.1 ± 13.7. The results of the Wilcoxon rank sum test and t-test revealed that there were significant differences in each dimension of the EQ-5D and in EQ VAS scores between the diabetes mellitus group and the group without diabetes. In all five dimensions, the proportion of subjects with no problems in the group without diabetes was higher than that in the diabetes mellitus group, and the proportion of extreme problems was lower. EQ VAS scores were also higher in older people without diabetes (*p* < 0.001).

**Table 2:** The results of the EQ-5D according to diabetes groups

Dimensions and levels	Diabetes mellitus N (%)/Mean ± SD	No diabetes mellitus N (%)/Mean ± SD	Total N (%)/Mean ± SD
Mobility			
no problems	1139(80.4)	8441(87.1)	9580(86.3)
some problems	235(16.6)	1112(11.5)	1347(12.1)
extreme problems	42(3.0)	134(1.4)	176(1.6)
p	< 0.001		
Self-care			
no problems	1248(88.1)	8968(92.6)	10,216(92.0)
some problems	120(8.5)	543(5.6)	663(6.0)
extreme problems	48(3.4)	176(1.8)	224(2.0)
p	< 0.001		
Usual activities			
no problems	1161(82.0)	8619(89.0)	9780(88.1)
some problems	190(13.4)	821(8.5)	1011(9.1)
extreme problems	65(4.6)	247(2.5)	312(2.8)
p	< 0.001		
Pain/discomfort			
no problems	1045(73.8)	7901(81.6)	8946(80.6)
some problems	345(24.4)	1701(17.6)	2046(18.4)
extreme problems	26(1.8)	85(0.9)	111(1.0)
p	< 0.001		
Anxiety/depression			
no problems	1293(91.3)	9060(93.5)	10,353(93.2)
some problems	114(8.1)	595(6.1)	709(6.4)
extreme problems	9(0.6)	32(0.3)	41(0.4)
p	0.002		
EQ VAS score	71.8 ± 14.8	76.8 ± 13.4	76.1 ± 13.7
p	< 0.001		

### The influence of diabetes mellitus on HRQoL adjusted for other characteristics

The results revealed the significant influence of diabetes and all the other factors on EQ VAS scores (Table 3, unadjusted model). The results showed that geriatric people who did not have diabetes and were younger were more likely to have higher EQ VAS scores. After adjusting for the influencing factors, diabetes mellitus was found to be significantly associated with EQ VAS scores (Table 3, multivariate model 1). The mean EQ VAS score of people who had diabetes mellitus was approximately 4.61 points lower than that of people who did not have diabetes (95% CI = -5.33,  -3.90, *p* < 0.001). The model was significant and explained 11.6% of the variance (F = 81.66, p < 0.001, adjusted R^2^ = 0.116). After considering hypertension and other chronic diseases (Table 3, multivariate model 2), people who had diabetes mellitus were approximately 3.70 points lower than people who did not have diabetes (95% CI = -4.40,  -2.99, *p* < 0.001). The model was significant and explained 17.3% of the variance (F = 117.46, p < 0.001, adjusted R^2^ = 0.173).

**Table 3:** Association of EQ VAS scores with diabetes and other factors

Variables	Unadjusted model		Multivariate model 1^a^		Multivariate Model 2^b^	
	B (95%CI)	p	B (95%CI)	p	B (95%CI)	p
Diabetes	−5.00(−5.76, −4.25)	< 0.001	− 4.61(− 5.33, −3.90)	< 0.001	−3.70(− 4.40, − 2.99)	< 0.001
Age	−4.99(− 5.31, − 4.67)	< 0.001				
Sex	− 1.84(− 2.35, − 1.34)	< 0.001				
Registered residence	−0.93(−1.57, − 0.29)	0.004				
BMI (kg/m^2^)						
< 18.5 vs. ≥28	−3.47(−4.85, −2.09)	< 0.001				
18.5–23.9 vs. ≥28	0.93(−0.12, 1.98)	0.081				
24.0–27.9 vs. ≥28	2.34(1.25,3.44)	< 0.001				
Education	1.10(0.86,1.34)	< 0.001				
Marital status						
Single vs. Married	−3.17(−5.31, − 1.02)	0.004				
Widowed vs. Married	−5.05(− 5.70, −4.40)	< 0.001				
Divorced vs. Married	0.43(−1.90, 2.77)	0.716				
Income source						
Oneself or mate vs. Other	5.60(4.56,6.64)	< 0.001				
Children or grandchildren vs. Other	−5.96(−7.25, − 4.67)	< 0.001				
Social relief vs. Other	−6.67(−9.09, − 4.26)	< 0.001				
Smoking	1.63(1.29,1.97)	< 0.001				
Drinking	3.65(2.98,4.32)	< 0.001				
Physical exercise	3.57(3.07,4.08)	< 0.001				
Health examination	1.64(1.12,2.16)	< 0.001				

^a^Model 1: Control variables including age, sex, registered residence, BMI, education, marital status, income source, smoking, drinking, physical exercise and health examination were entered in the model

^b^Model 2: Model 2 is multivariate model 1 plus hypertension and other chronic diseases

**Fig. 1:**
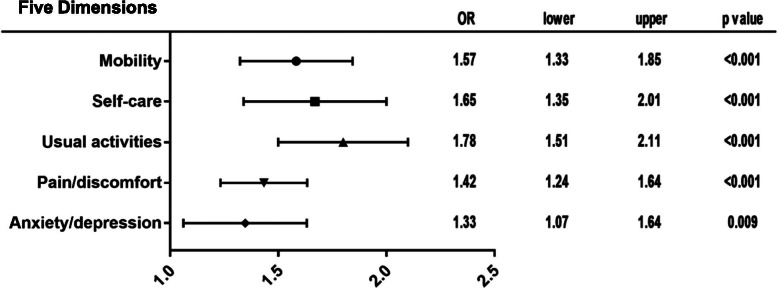
Forest plot of the five dimensions of the EQ-5D between people with and without diabetes mellitus*. *Adjusted for age, sex, registered residence, BMI, education, marital status, income source, smoking, drinking, physical exercise, health examination, and hypertension and other chronic diseases

People with diabetes mellitus were more likely to have problems across the five dimensions of the EQ-5D. They had increased problems with mobility (OR = 1.57, 95% CI = 1.33,1.85), self-care (OR = 1.65, 95% CI = 1.35,2.01), usual activities (OR = 1.78, 95% CI = 1.51,2.11), pain/discomfort (OR = 1.42, 95% CI = 1.24,1.64), and anxiety/depression (OR = 1.33, 95% CI = 1.07,1.64) (Fig. 1).

## Discussion

The results of the analysis demonstrated that there were significant differences in the five dimensions (mobility, self-care, usual activities, pain/discomfort and anxiety/depression) of the EQ-5D and in EQ VAS scores between older people with or without diabetes mellitus. The EQ VAS scores of people who had diabetes mellitus were approximately 3.70 points lower than those of people who did not have diabetes (95% CI = -4.40,  -2.99, *p* < 0.001) after adjusting for confounding variables. The older people with diabetes mellitus in this study indicated that they were aware of their disease and had been diagnosed by a doctor.

The strength of the study is that it is a randomised survey of a large sample, and the results are reliable and can reveal the association between diabetes mellitus and HRQoL. The limitation of the study is whether diabetes mellitus was reported by the older participants, which means that some diabetes patients were unlikely to know they had diabetes and had not been diagnosed by a doctor, which led to a lower incidence rate of diabetes in the study. However, there was no measurement of glucose values or haemoglobin A1C in the original design. Additionally, because the survey database removed private information, we could not sample some of the subjects to measure their glucose values or haemoglobin A1C to estimate the extent of undiagnosed diabetes. This limitation may lead to underestimation of the prevalence of diabetes in older people.

Previous studies on the associations between diabetes and some aspects of quality of life in older people showed relevant findings, and the current study provides more updated results. People with diabetes were more likely to have problems with mobility because of the complications of chronic disease, which even led to disability [16]. Similar findings were reported in a study that carefully measured physical mobility, including running, lifting weight, or walking approximately 100 m [17]. A previous studies concluded that diabetes patients experience limitations in mobility tasks, general physical activities, and leisure and social activities [18], but few studies have investigated the relationship between diabetes mellitus and self-care or usual activities. Pain or discomfort is more common in people with diabetes, but there was a sex difference not detected in our study. Women with diabetes experienced more frequent and intense pain than women without diabetes, and pain was often felt in the shoulders, knees, and upper extremities; however, the corresponding difference was not observed in men [19]. The injection of insulin also caused mild discomfort in diabetes patients [20]. A meta-analysis showed that people with diabetes often have back pain, but a direct causal link was not established [21]. There are also studies about the association between diabetes and anxiety or depression. A meta-analysis indicated an association between anxiety and diabetes [22], and depression often co-occurs with diabetes [23]. There was also a sex difference in that the effects of mental health symptoms appeared to be stronger in men with diabetes than in women [24]. The EQ VAS scores of older people with diabetes mellitus were lower than those of people without diabetes, which means that the geriatric population with diabetes had a lower HRQoL. A study on diabetes patients who were 18 years and older showed a negative effect of diabetes on EQ VAS scores [25], and a similar conclusion was made in a Vietnamese study, indicating that older diabetic outpatients had lower EQ VAS scores (57.5 ± 14.4) [26]. In our study, the mean EQ VAS score of older people with diabetes was 71.8 ± 14.8, which was much higher than that in the Vietnamese study. One possible reason is that in the Vietnamese study, the participants were outpatients who may suffer more from the disease, and our study was community-based. Another possible reason is that Shanghai has a more advanced economy and medical care than Vietnam, and 80.3% of the interviewees were urban residents, so older people with diabetes in Shanghai have a better HRQoL than those in Vietnam.

The present study demonstrated that diabetes mellitus does have a negative impact on the HRQoL of older people in Shanghai across all five dimensions of the EQ-5D. Emerging evidence highlights the adverse impact of diabetes mellitus on the HRQoL of older people, and the management of diabetes mellitus seems to be important for improving the HRQoL of the geriatric population. There was promising improvement in the management of diabetes mellitus, such as proper care, adherence to diabetes guidelines and screening for diabetes-related complications [27]. Older people with diabetes may achieve a better HRQoL by controlling glycaemia and preventing late-term complications, and the government can promote a heathier geriatric population by improving health care system performance.

There could be more influencing factors that were not considered and included in the analysis due to the original survey design, which should be taken into account in further research. Moreover, the glucose values or haemoglobin A1C could be measured to include the currently undiagnosed diabetic people to the diabetes population to obtain a more accurate estimate of how large the gap in EQ VAS scores is between diabetic people and nondiabetic people.

## Conclusions

This study showed that diabetes mellitus was associate with the HRQoL of older people in Shanghai and that older people with diabetes had poorer performance in every aspect of EQ-5D measurements. They were more vulnerable to problems with mobility, self-care, usual activities, pain/discomfort and anxiety/depression and had lower EQ VAS scores. The older population with diabetes mellitus should be given more attention by individuals and medical providers to achieve a better HRQoL.

## Data Availability

The data that support the findings of this study are available from the Centre for Health Statistics and Information of Shanghai, but restrictions apply to the availability of these data, which were used under licence for the current study and so are not publicly available. Data are, however, available from the authors upon reasonable request and with permission from the Centre for Health Statistics and Information of Shanghai.

## Abbreviations

HRQoL: Health-related quality of life
EQ-5D: European Quality of Life-5 Dimensions
EQ VAS: European Quality of Life Visual Analogue Scale
CI: Confidence interval
OR: Odds ratio
TIA: Transient ischaemic attack
RA: Rheumatoid arthritis
NHHS: National Household Health Survey
BMI: Body mass index
SD: Standard deviation
